# The hepatitis C infection in Iran: a policy analysis of agenda-setting using Kingdon’s multiple streams framework

**DOI:** 10.1186/s12961-019-0436-z

**Published:** 2019-03-27

**Authors:** Masoud Behzadifar, Hasan Abolghasem Gorji, Aziz Rezapour, Nicola Luigi Bragazzi

**Affiliations:** 1 Health Management and Economics Research Center, Iran University of Medical Sciences, Tehran, Iran; 2 School of Public Health, Department of Health Sciences (DISSAL), University of Genoa, Genoa, Italy

**Keywords:** Hepatitis C, Iran, agenda-setting, Kingdon’s multiple streams framework, health policy

## Abstract

**Background:**

Hepatitis C virus (HCV) infection causes a large number of deaths annually worldwide. Policies play an important role in regulating healthcare agendas and prioritising of health-related issues. Understanding these priorities is very important in health. The objective of this study was to investigate HCV-related issues and their influence on agenda-setting in Iran.

**Methods:**

A qualitative design was used. Data were collected by carrying out a review of documents and interviews. A comprehensive search was conducted to identify documents related to HCV-related policies in Iran. Semi-structured interviews were conducted with both purposive and snowball sampling of 14 interviewees related to the HCV programme in Iran, including government officials, civil society, development partnership members and academicians. Documents and interview data were analysed manually and using MAXQDA Version 10 software. Kingdon’s multiple streams framework was used to guide data analysis.

**Results:**

The factors which influenced HCV-related agenda-setting were lack of proper information of the HCV epidemiology before the 1990s, lack of diagnostic facilities, neighbouring countries with high HCV prevalence, the stigma of HCV, high prevalence in prisoners, international evidence and high costs generated by HCV. The factors related to policy were effective treatment methods, drug production inside Iran, Iran Hepatitis Network, support outside government group elites and academicians. The factors related to political will were international influence, changes in the government and parliament support.

**Conclusion:**

The findings of this study showed that there are various national and international factors that play a role in shaping HCV-related policies. It seems that, if HCV is put into the agenda, it can be eliminated in Iran by 2030 by supporting and implementing appropriate programmes from decision- and policy-makers.

## Background

Hepatitis C virus (HCV) infection causes a large number of deaths annually worldwide [1] and has become one of the key healthcare challenges [2]. It is estimated that 1% of the world’s population (approximately 71 million people) is infected with HCV [3]. Despite the introduction of direct-acting antiviral drugs in different countries, with preliminary promising results, the number of deaths due to this infection continues to increase [4]. The high cost of the drugs used, the limited capacity of laboratories and other facilities, the lack of financial resources in the health sector and the lack of support for many policy- and decision-makers in low- and middle-income countries represent some major barriers to the proper access to treatment of patients with HCV [5].

Various countries worldwide have implemented appropriate policies to reduce the incidence and mortality of HCV, whilst designing and developing plans to control this public health problem in the years to come [6–8]. To control HCV, effective policies are needed that require the efforts of international, regional and national stakeholders. Participation of these stakeholders creates a high political commitment and mobilises all resources to implement these policies [9, 10].

Advocacy in HCV control programmes has led to achievement of effective patient treatment and management goals as well as a reduction in the costs of medicines through the establishment of pharmaceutical companies to produce medicines locally overcoming the need to buy them from third countries [2]. Additionally, advocacy of the country’s stakeholders is an important and vital element integral to healthcare policy [11]. To prevent, control and cure a disease, new policies need to be implemented or existing policies must be changed [12], both of which are very complex policy processes [13]. Policy- and decision- makers decide on the various issues affecting the health sector, including the amount of personal interest, the magnitude of the topic, the available scientific evidence, political and social considerations, the efforts of interest groups and their impact on the topic. All these issues can influence the decision of health policy- and decision-makers of whether to place a topic on their agenda [14]. Thus, various factors can impact on the process of agenda-setting and should be taken into consideration [15].

Existing policies to control HCV can accelerate the process of reducing, and even eliminating, its burden if they are taken seriously by policy- and decision-makers and put on their agenda [16]. When a major issue, such as HCV, is put into action, comprehensive support for the issue will make policies work properly; thus, the role of policy- and decision-makers in the success or failure of an important policy is of crucial importance [17]. For an issue like HCV to become a priority, complex processes are undertaken; the priority of a policy or programme is defined by the degree of policy- and decision-makers’ attention to financial, human, and technical issues and resources that can be used or mobilised to implement that policy [18].

HCV in Iran is an important public health challenge and should be addressed by health policy- and decision-makers. Despite the low prevalence of HCV in the general Iranian population, the infection is on the rise [19]. It is expected that, in the near future, this infection will be the most important cause of deaths related to hepatitis in Iran [20]. Its high prevalence is particularly alarming in high-risk groups such as injecting drug users (IDUs) and prisoners [21, 22]. Undoubtedly, HCV should be placed on policy- and decision-makers’ agenda. Policies play a vital role in health issues related to community health, and therefore understanding the relevant factors that can impact on the process of agenda-setting is very important.

The present investigation aimed to better understand HCV-related issues and their impact on policies in Iran. To achieve this, Kingdon’s multiple streams framework was deemed suitable for the purpose and, as such, was selected for this study. This framework is an appropriate technique for the planned analysis in that it enables the exploration of HCV-related issues and the effects they have on the process of agenda-setting. Being this the first analysis on HCV-related issues and their impact on policies in Iran, the investigation was conducted at a macro-level (national level), rather than focusing on a single region or province.

### The theoretical framework

Kingdon’s multiple streams framework is one of the most used conceptual tools for understanding the process of policy-making, including policies in the field of healthcare. This instrument enables scholars to capture the different steps of the policy-making process, including how policies are developed and implemented and which are the obstacles (lack of interest, lack of clarity and ambiguity, among others) that hinder the full implementation of a given policy. In detail, Kingdon’s framework includes three elements or independent streams. Providing appropriate conditions for an interaction among these three issues will cause opportunities to be created and will facilitate the process of agenda-setting by policy- and decision-makers [23]. As previously mentioned, this framework can be used to explain how different policies have addressed various topics in the health sector [24–32].

One of these streams is the ‘problem’ stream. This stream includes indicators, focusing events or feedback mechanisms. Indicators include issues that health policy- and decision-makers, researchers, doctors, and other stakeholders can achieve in their daily routine monitoring activity or scientific research. Policy- and decision-makers can use these indicators to determine the impact of a topic and evaluate different scenarios about them. Some examples of these indicators include the outbreak of a disease, the rate of death from road accidents, the shortage of manpower and increased healthcare costs. Focusing events such as a tragedy, a crisis or a disaster can trigger policy- and decision-makers’ feedback on a given issue. Many indicators alone cannot determine the importance of an issue, which is rather given by the complex interactions among indicators. Within Kingdon’s framework, several actors, such as government officials, the media, interest groups, industries, legislators and constituents, as well as other stakeholders, play a major role.

The second of Kingdon’s streams is the ‘policy’ stream. This stream involves potential solutions to the problem and allows individuals and groups to work on a solution to further develop it. Actors involved in this process can include lawmakers, special interest groups, policy entrepreneurs, professionals, academicians and industry researchers. These people can provide different alternatives or problems, issues and situations in order to influence the process of agenda-setting. Factors such as the level of support for individuals and groups, costs, industries and past experiences can also influence the choice of alternatives.

The final stream in Kingdon’s model is the ‘political’ stream*.* National mood, shifts in administration, media influence, interest group advocacy campaigns, elections and ministerial changes can have an impact on the process of policy- and decision-making. In addition, legislators and administrators also have an influence on political processes.

Based on Kingdon’s model, the interaction of these three streams determines the process of agenda-setting. Policies are implemented effectively when all of these streams converge, resulting in a policy window. Policy entrepreneurs can facilitate this process and create opportunities for policy progress. As previously stated, the aim of this study was to investigate the determinants of the process of agenda-setting of HCV-related issues in Iran.

## Methods

### Data collection

Data were collected by carrying out a comprehensive review of relevant HCV-related documents and interviews.

### Document analysis

A comprehensive search was conducted to identify documents related to HCV issues and policies, contained in scholarly publications as well as in the grey literature in Iran. The websites of the different pertinent ministries and related organisations, such as the Ministry of Health and Medical Education (MoHME), the Parliament (*Majlis*), the Iranian Judicial system, the Ministry of Cooperatives, Labour and Social Welfare, the State Prisons and Security and Corrective Measures Organisation, the Red Crescent, the Imam Khomeini Relief Foundation, and the Ministry of Sport and Youth, were consulted. Additionally, scholarly databases, including PubMed/MEDLINE, ISI/Web of Science, EMBASE and Scopus, were searched from January 1990 up to July 2018, using a string of keywords related to the topic under study. Google Scholar was used to retrieve the grey literature. Concerning the search strategy, the following keywords were selected: (policy OR policies OR plans OR programs OR strategies OR solutions) AND (‘hepatitis C virus’ OR ‘viral hepatitis’ OR HCV) AND Iran.

National guidelines, training manuals and handbooks, health reports, national health plans, international, national and regional policies, health protocols, non-governmental organisation (NGO) reports, and HCV-related laws were also searched; this search was done independently by two authors in order to identify all documents.

### Semi-structured interviews

Semi-structured interviews were conducted with both purposive and snowball sampling of 14 interviewees related to the HCV programme in Iran, including government officials, civil society, development partnership members and academicians. These people were involved in the planning, decision-making, policy-making and support of HCV programmes.

Interviews were digitally recorded and saved using a tape recorder. During the interview, a handwritten note was also prepared. The interview time was between 45 and 70 min. Interview data accuracy was achieved by reviewing the handwritten texts by interviewees and using their complementary comments as well as involving well-trained researchers in the process. In addition, two experts in the field of qualitative studies monitored the whole interview process. The use of an integrative method, including note-taking during interviews and a sampling method ensuring maximum diversity, provided data reliability and transferability [33].

With regards to policies concerning HCV, the following questions were asked:

1. Problem stream: What challenges still unresolved and unaddressed make HCV a problem in Iran?
2. Policy stream: What solutions have been found and adopted by individuals or groups to address HCV-related issues in Iran?
3. Political stream: What are the political factors that can influence the effective adoption of HCV-related policies in Iran?

## Analytical approach

Data extracted from documents and interviews were analysed both manually and using MAXQDA Version 10 software for content analysis. Following familiarisation with the data and a preliminary analysis of the interviews, initial codes were prepared and, after removing the same duplicate codes, they were interpreted and finally assigned under the themes of Kingdon’s multiple streams framework (problem, policy and political streams). The principles of content analysis were applied to guide and inform the data analysis.

## Ethics

Written, informed consent was obtained from each participant after a clear and thorough explanation of the study objectives and assurance of the confidentiality of one’s own identity. Ethical approval for this study was obtained from the authors’ institute prior the commencement of the investigation.

## Results

According to Kingdon’s framework, the effective factors associated with HCV-related issues and policies, which can be described as a priority in Iran, can be analysed and subdivided into the problem, policy and political streams.

### Problem stream

#### Lack of proper information on HCV before the 1990s

The participants stressed that, until the 1990s, there was not enough information on HCV in Iran concerning the number of patients with HCV and the epidemiological and clinical burden of the disease. Indeed, no attention was paid to this disease, and remained inadequately addressed by the healthcare departments and institutions. Only after the advent of contaminated blood, HCV began to be considered as an issue by policy- and decision-makers.

#### Diagnostic facilities

The respondents stated that, in the same years, laboratory facilities were limited in their ability to diagnose the disease due to the costs, and therefore many people were not able to identify their illness due to lack of diagnostic facilities.

#### Neighbouring countries with high prevalence of HCV

The participants stated that the prevalence of HCV in Iran is lower than in its neighbouring countries, such as Iraq, Afghanistan, Pakistan, Turkey, Azerbaijan and Tajikistan, which have a high HCV prevalence rate. The movement of people from these countries to Iran, especially those at a higher risk, could cause the transmission of new cases.

#### Transit from Afghanistan and increased number of addicts

The respondents declared that Afghanistan, the largest producer of narcotics in the world and one of Iran’s neighbouring countries, is one of the main trafficking routes. Hence, the number of IDUs in Iran has increased and, according to the available scientific evidence, the prevalence of HCV among these individuals is high and has raised serious concerns.

#### HCV and stigma

The respondents stressed that, for cultural and religious issues, the stigma of HCV is high in Iran. Due to low awareness among people about HCV, many of them consider HCV-infected individuals as HIV-positive, which has isolated patients and made them refrain from pursuing their diagnosis and treatment. People with HCV have fear and anxiety, and are not willing to collaborate with physicians and be treated because they feel blamed for their condition.

#### High HCV prevalence in prisoners

The respondents stressed that the various studies have shown that HCV levels in prisoners are higher than in other populations in the community. The high-risk behaviours that exist in prisons can provide the basis for the high prevalence of this disease. On the other hand, prison officials, for various reasons, are not willing to cooperate fully with diagnostic and therapeutic procedures, and they have problems with providing prisoners with syringes.

#### International evidence

The participants stated that, in its 2017 report on hepatitis, WHO announced that the Middle Eastern region, including Iran, has a low-moderate HCV prevalence, except for some areas with high levels of HCV infection. However, according to this report, the death rate from hepatitis has increased between 2005 and 2015, and is alarmingly high for Iran.

#### High costs of HCV

The respondents stressed that the costs generated by HCV treatment and management are high and many patients are not able to pay for them. The MoHME’s funding for this disease is limited and therefore the lack of attention to these patients causes serious problems for them and other people in the community. The MoHME alone cannot afford all HCV-related costs. If patients are not supported, they will avoid treatment, causing many people at risk of contracting HCV to actually contract it.

#### Increased HCV in IDUs

IDUs are considered worldwide as one of the most important high-risk groups for HCV infection. They represent an alarming issue for all countries, including the health sector in Iran.

### Policy stream

To combat HCV, various alternatives can be used in the areas of prevention, screening and treatment. Hence, these alternatives can help policy- and decision-makers to find ways to eradicate HCV in their country.

#### Effective treatment methods

The participants stressed that, in recent years, drug treatments for HCV have led to an effective option for managing this disease, with many countries having used available therapeutic solutions for eradicating this disease. Support from the MoHME in Iran provided the basis for the treatment of these patients. Establishing a diagnostic network in governmental laboratories and delivering patient medications would provide a way for better interventions to improve the status of HCV patients.

#### Drug production inside Iran

The interviewees declared that the support of pharmaceutical companies plays an important role in the process of treating and managing HCV patients. Expensive drugs necessary for curing this disease can be a barrier to the treatment of patients. Therefore, pharmaceutical companies in Iran, with private and government sector investments, have started producing HCV drugs and significantly curbing their prices.

#### Iran Hepatitis Network (IHN)

The respondents stressed that individuals and groups at home and abroad supported HCV control programmes. The IHN, a government-affiliated entity, consists of specialised physicians, laboratory staff and experts in the field of HCV providing annual training, identification and treatment of HCV patients, and screening of people at a higher risk with high levels of protection. The IHN’s activities are unique – the IHN has been able to offer prevention and disseminate high-quality information. It has also been able to convince many political groups in the community that HCV should be given more attention. People in this network all have a high level of expertise in HCV-related areas.

#### Support outside government groups

The interviewees stated that the NGOs and community-based organisations play an important role in preventing crime and social exclusion. NGOs represent a social capital for governments. These NGOs have advocated HCV prevention policies in recent years. Various campaigns that have led to increased awareness among the general population have, to some extent, contributed to the improvement of information on the disease. Campaigns in schools, universities, drug addiction centres and parks organised by these NGOs have raised awareness of HCV. Moreover, with the participation of the people, stigma towards HCV patients has decreased.

#### Elites and academicians

The respondents stressed that elites and academicians doing research in the field of HCV can be very effective in prioritising the disease. Professor Reza Malekzadeh is Deputy of Research and Technology of the MoHME in Iran; he has been the minister of health in previous years and is one of the most renowned academic figures in Iran and the world. His views on specialised committees such as the Hepatitis Committee and meetings with other organisations for HCV are important and can therefore influence their decisions. He has been able to encourage pharmaceutical companies to produce HCV drugs and he also plays a valuable role in providing scientific evidence and research studies.

#### The judicial system supports harm reduction programmes in prisons

The Iranian judicial system has effectively supported harm reduction programmes among prisoners and has paved the way for the implementation of other ad hoc prevention programmes.

### Political stream

#### International influence

The respondents stressed that the 2015 Sustainable Development Goals (SDGs) played an important role in emphasising the fight against HCV, and one of the goals to eliminate HCV has been identified by the year 2030. The SDGs have made Iran’s policy- and decision-makers to be willing to commit to achieving the goal of eliminating HCV by 2030.

#### Changes in the government

The respondents stressed that, with the start of the government of President Rouhani, health promotion was introduced as one of his most important campaign slogans. In 2014, the Health Transformation Plan started with a variety of goals. Over time, different goals have been considered, introduced and gradually achieved. Additionally, the control of infectious diseases such as HCV was a serious target. With the launch of this plan, health was seen as a serious priority among Parliament and decision- and policy-makers in Iran. Appropriate funding was allocated to the plan, leading to the development of less advanced areas that did not have a good health status.

#### Parliament support

The participants stated that, when matters are considered by the Parliament, the required rules are passed faster and more accurately. Certified people at the Parliament’s Health Commission try to find new sources of funding for health-related issues, including HCV, in order to achieve better health and reduce mortality among members of the community. Individuals in Parliament in many cases can help to improve the health status of the community and can change the cultural perspective of society with new and appropriate laws.

## Discussion

Policies play an important role in regulating the process of agenda-setting and prioritising of health-related issues. Understanding these priorities is very important in the field of healthcare. The use of Kingdon’s multiple streams framework in this study helped us to examine the different dimensions and factors of HCV-related issues based on three streams (namely, problem, policy and political will) in Iran.

The results showed that, if properly understood and effectively utilised, the use of conceptual frameworks, such as Kingdon’s theoretical tool, can provide an appropriate and deep understanding of the impact of several health-related issues on policy- and decision-makers.

### HCV epidemiology in Iran

Indicators in the public and private sectors can be utilised as warnings for policy- and decision-makers. Existing evidence and scholarly studies on the epidemiology of HCV in Iran indicate that the incidence/prevalence rate of this disease is not high yet many people with this condition are not aware of their infection state [34, 35]. Furthermore, due to the easy transfer within neighbouring countries, the transmission of various diseases has made health policy- and decision-makers more aware of the disease with regards to the specific regional situation [36]. People from various neighbouring countries to Iran undergo numerous trips to some of Iran’s religious cities. Many Afghans entered the Sistan and Baluchestan province via the Eastern border of Iran, and given the higher prevalence of HCV in Afghanistan, there is a risk of transmission and contagion from these travellers. In the past, there have also been some similar experiences with viral hepatitis [37].

### HCV drugs and related costs

Health policy- and decision-makers look for cost-effective alternatives since HCV treatment, prevention and management can be costly for the country’s health system [38]. The existence of effective HCV therapeutics able to eradicate the virus should stimulate policy- and decision-makers to find solutions in order address this disease in a sustainable way [39]. The cost of HCV treatment is indeed high and, according to the global guidelines and suggestions from health institutional bodies, the HCV drugs should be included in the list of essential therapeutics [40]. Many patients are not able to start or complete their treatment due to a lack of financial support from the health departments and lack of insurance coverage [41]. Policy- and decision-makers in Iran stated insufficient funding for the provision of drugs and medicines to treat HCV patients. Furthermore, medications were limited and the Ministry of Health was confronted with important limitations regarding the availability of drugs. Drug production inside a given country can reduce local drug prices. Indeed, Egypt’s experiences in drug production and the subsequent reduction of drug prices are a good example to follow [42]. Further, encouragement and support from the Iranian Ministry of Health has raised interest from pharmaceutical companies to produce drugs within Iran, with a subsequent reduction in the cost of these medications. Additionally, insurance companies are also keen to cover the cost of drugs and treatment for patients since this is significantly lower than before.

### Involvement of stakeholders in the process of decision- and policy-making

The role of policy communities as groups that involve individuals with expertise in a field is important in providing ideas and innovations in the field of healthcare policies [43]. IHN consists of specialised network of people who play a very important role with regards to HCV-related issues. The presence of people who, in the past, have served as important managers in the MoHME’s network can provide good ideas for enriching, developing and effectively implementing HCV-related programmes.

Disagreements between associations and groups often occur when discussing various issues; however, in Iran, given the importance of controlling this disease, good convergence has been established between many groups, NGOs and the IHN. Indeed, various groups and NGOs in Iran are trying to focus on HCV issues regardless of their own interests, with integrity and team work to achieve the elimination of HCV. According to Kingdon’s model, one of the destructive effects of fragmentation between groups is insecurity and instability [23], and lack of consensus due to different orientations [44]. However, despite the diversity of groups involved in the treatment or management of various diseases, including HCV, their activities are being carried out in pursuit of the same goal – the fight against HCV. Further, there can be many actors in relation to a policy [45]; these actors can be either inside or outside government. Within the government, employees of the Ministry of Health, government groups and organisations give their support for the elimination of HCV, and outside the government body, private groups and companies have agreed to implement HCV control programmes [46].

The convergence of different stakeholders leads to more attention from policy- and decision-makers [47]. Although people in the various groups have different goals, if an important issue such as HCV, the elimination of which is critical for the overall community health, is on the agenda, this convergence can be valuable [48].

### Global actors and their influence on HCV-related policies in Iran

The findings of this study presented some of the influential factors in the political stream impacting on HCV elimination policy in Iran; globalisation is one of these factors [49]. The Millennium Development Goals experience and commitment by countries to achieve them in 2015 showed that policy- and decision-makers from various countries are keen to increase the quality of life for people in terms of health indicators [50]. Unfortunately, the Millennium Development Goals did not focus much on HCV, likely leading to the increasing epidemiological burden (prevalence/incidence rate) of the disease in the past years. Conversely, all countries within the SDGs have pledged to eliminate HCV by 2030 [51].

### Local political events and their influence on agenda-setting

Elections and changes in government are among the most important political events that can have meaningful effects in prioritising a given policy [52, 53]. The new Iranian government, which was settled in 2013, announced healthcare as one of its slogans. After winning the election, health policy- and decision-makers enforced a healthcare reform. In the first step, the goal was to reduce people’s out-of-pocket expenses and to spread health equity [54]. Over time, various programmes have been implemented in this reform, and the increase of access to health services in less developed regions and high-risk individuals has also been of interest to policy- and decision-makers; programmes to control various infectious diseases were also included in this package. The most important part of this reform was the allocation of new financial resources for health. Therefore, policy- and decision-makers, with more financial resources, tried to expand their financial support for different patient groups, with HCV seemingly receiving more attention in light of the new conditions [37]. As individuals are involved in the decision- and policy-making process, they usually support certain policies, creating coalitions and striving to prioritise given policies [23]. The existence of influential people can facilitate the process of agenda-setting since their position allows them to convince policy- and decision-makers to focus on a given policy [55]. In the Iranian Parliament, certain individuals can convince other decision- and policy-makers to focus on issues such as HCV. In recent years, the Iranian parliament has supported health sector programmes and has adopted good laws.

### Window of opportunity and policy entrepreneurs

According to Kingdon’s model, if all the three issues of problem, policy and political will are linked, the likelihood of an issue being on the agenda is very high [23]. Thus, the ‘window of opportunity’ for HCV appears to be open in Iran. Policy-makers have different and appropriate evidence to take advantage of this opportunity window.

When the goal of HCV elimination by 2030 was set as a target by WHO in the SDGs, a new opportunity window was created. WHO, with the cooperation and commitment made by all countries and through the cooperation of all stakeholders, has made many efforts towards the achievement of this goal. With the implementation of the Health Transformation Plan, the Iranian government has begun to prioritise health-related issues and HCV has also been of interest both to decision- and policy-makers.

The documentation on infectious diseases has indicated that Iranian decision- and policy-makers are, indeed, interested in controlling HCV. On the other hand, not all stakeholders have shown the same level of interest and commitment towards the development and implementation of some HCV-related policies.

The interviewees stated that the most important HCV-related actor, the MoHME, is acting as a policy entrepreneur, but needs the support of all the other stakeholders.

### Comparison with other studies

The existing scholarly literature focusing on policy processes in low- and middle-income countries analysed by means of Kingdon’s multiple streams framework is rather limited. Few studies have explored such topics. A recently published investigation, conducted in Kenya [56], addressed some of the major health challenges this country is experiencing and the obstacles hindering the full implementation of intersectoral collaborations and actions focusing on health promotion. In Iran [57], a qualitative case study approach was employed to address the ‘Health in All Policies’ programme; Kingdon’s model was utilised at the micro-level (province level) and political commitment and policy entrepreneurs were found to be the two most relevant leverages in the political process. In Cameroon [58], the policy analysis of performance-based financing in the healthcare sector showed the importance of setting a dedicated team in order to implement and pursue the policy. The process of decision- and policy-making was also influenced by other factors, including available media information, scientific evidence, and previous policies and experiences.

Other studies are from developed countries, such as Ireland [59], Canada [60] and United Kingdom [61]. In Ireland [59], Kingdon’s multiple streams theory has been used to analyse and provide recommendations regarding diabetes care. Authors were able to describe the non-convergence of the politics stream due to the lack of support from health service management, changes at the level of the organisational structure and lack of adequate funding. In Canada [60], the theoretical framework was used to investigate HPV vaccination-related issues, underlining how the existence of cost-effectiveness models and advocacy of stakeholders, including citizens and HPV-affected politicians, influenced the government in deciding to fund the immunisation campaign also for male subjects. In the United Kingdom [61], using this model, the author was able to uncover continuities and discontinuities in the problem, politics and policy streams over the past 70 years of the British National Health Service.

All these studies have found Kingdon’s multiple streams tool to be informative and useful in providing an accurate analysis reflecting the different stages and steps of decision- and policy-making, in terms of opportunities, obstacles and challenges.

### Strengths and limitations

This study has some strengths and limitations that should be properly acknowledged. The strengths include the use of a well-known conceptual framework (Kingdon’s multiple streams model), which enables scholars to collect relevant data and to perform an accurate analysis of HCV-related issues and their impact on policies in Iran. Kingdon’s model is, indeed, a valuable structured tool, which focuses on the different aspects of the processes of policy- and decision-making. Besides methodological rigor and transparency, another strength of the present investigation is given by its novelty, in that it explores a topic usually overlooked in the existing scholarly literature. There is, indeed, a dearth of data and information about healthcare-related decision- and policy-making in low- and middle-income countries.

On the other hand, this study suffers from some limitations. For instance, the sample of participants was not completely representative of the entire civil society. Moreover, the study design was cross-sectional and not longitudinal. Finally, the focus of this article was national and not regional, and it failed to address the cultural and social differences that may exist in different provinces of Iran.

### Future prospects

Based on the above-mentioned shortcomings, further research is urgently needed in the field. Future studies should employ a larger sample size, perhaps utilising a randomised sampling technique, and a longitudinal study design in order to capture time trends and temporal patterns. Additionally, a regional focus should be adopted in order to collect relevant actionable data useful for policy- and decision-makers at the meso- and micro-levels.

## Conclusion

The present study examined HCV-related issues as a priority for Iran’s healthcare system using Kingdon’s multiple streams framework. The findings of this investigation showed that there are various national and international factors that may play a role and influence the process of agenda-setting. The epidemiology of this disease as a global challenge, the support of various groups and the existence of political changes in Iran are among the factors that have led HCV to be considered a priority. It can be anticipated that, if HCV is put on policy- and decision-makers’ agenda, HCV can be eliminated in Iran by 2030 by supporting and implementing appropriate policy programmes.

## Abbreviations

HCV: hepatitis C virus
IDU: injecting drug users
IHN: Iran Hepatitis Network
MoHME: Ministry of Health and Medical Education
NGO: non-governmental organisation
SDGs: Sustainable Development Goals
